# Reducing Spread of Infections with a Photocatalytic Reactor—Potential Applications in Control of Hospital *Staphylococcus aureus* and *Clostridioides difficile* Infections and Inactivation of RNA Viruses

**DOI:** 10.3390/idr13010008

**Published:** 2021-01-11

**Authors:** Abeer Gharaibeh, Richard H. Smith, Michael J. Conway

**Affiliations:** 1 Insight Research Institute, Flint, MI 48507, USA; Richard.smith@iinn.com; 2 Department of Research, Insight Surgical Hospital, Warren, MI 48091, USA; 3 Insight Research Center, Insight Institute of Neurosurgery & Neuroscience, Flint, MI 48507, USA; 4 Foundational Sciences, College of Medicine, Central Michigan University, Mt. Pleasant, MI 48859, USA

**Keywords:** *Staphylococcus aureus*, MRSA, *C. difficile*, photocatalytic reactor, photocatalytic oxidation, infection control, RNA virus, dengue virus, coronavirus, COVID-19

## Abstract

Contaminated surfaces and indoor environments are important sources of infectious spread within hospital and non-hospital facilities. Bacterial infections such as infections with *Clostridioides* (formerly *Clostridium*) *difficile* (*C. difficile*) and *Staphylococcus aureus* (*S. aureus*) and its antibiotic resistant strains continue to pose a significant risk to healthcare workers and patients. Additionally, the recent emergence of the coronavirus disease 2019 (COVID-19) pandemic, which is caused by the novel coronavirus, severe acute respiratory syndrome coronavirus 2 (SARS-CoV-2), highlights the need for safe and effective methods to decontaminate surfaces to control infection spread in hospitals and the community. To address these critical needs, we tested a photocatalytic reactor decontamination method to disinfect contaminated surfaces in a hospital and a laboratory setting. By placing the reactor in a test hospital room, growth of *S. aureus* and *C. difficile* were significantly reduced compared with a control room. Additionally, using a model enveloped positive-sense single-stranded RNA virus, dengue virus type 2 (DENV2), we showed that the use of the photocatalytic reactor reduces viral infectivity. Collectively, the results demonstrate the potential utility of photocatalytic reactors in reducing the spread of highly contagious bacterial and viral infections through contaminated surfaces and environments.

## 1. Introduction

The discovery and implementation of novel environmentally friendly technologies for infection control are critically needed [1]. The emerging trends in healthcare-associated infections (HAIs) and the community spread of highly transmissible diseases, such as the new coronavirus disease 2019 (COVID-19), necessitates the need for easy-to-use, safe, and economic technologies to prevent the spread of diseases among patients, hospital workers, and in the community [2]. Contaminated surfaces and environments are important sources for the transmission and spread of infections including those caused by *Clostridioides difficile* (*C. difficile*), methicillin-resistant *Staphylococcus aureus* (MRSA), vancomycin-resistant enterococci (VRE), norovirus, and the emerging novel coronavirus, severe acute respiratory syndrome coronavirus 2 (SARS-CoV-2), which causes COVID-19 [1]. One-day surveys of 11,282 inpatients in 183 hospitals in the US found that 452 of them had one or more HAIs [3]. Pneumonia and surgical site infections were the most common, with each accounting for 21.8% of cases, followed by gastrointestinal infection which constituted 17.1% of cases. *C. difficile* was the most common causative organism, followed by *Staphylococcus aureus (S. aureus)*, and then *Klebsiella pneumoniae, Klebsiella oxytoca*, and *Escherichia coli* (*E. coli*) [3]. In addition to the health impact, HAIs cause an economic burden with an estimated annual medical cost in the US ranging from $28.4 to $33.8 billion [4].

Improved control and reduction of HAIs are perpetual concerns in hospitals and other healthcare settings, including non-hospital residential facilities such as nursing homes and correctional facilities [5,6,7]. Control of *C. difficile* infection is considered a major challenge in healthcare facilities due to the capability of *C. difficile* spores to survive on hard surfaces for up to 5 months and their resistance to traditional environmental cleaning [8]. A 10-year review performed on *C. difficile* patients in acute care hospitals in the US during 2005 to 2014 indicated that the incidence of *C. difficile* infection is increasing [9]. It is estimated that half a million *C. difficile* infection cases occur yearly in the US with more than 24% of the cases are in hospital settings [10]. Another study found that 82% of patients with community-associated *C. difficile* infection reported that they had a history of outpatient or inpatient healthcare exposure without an overnight stay [11]. *S. aureus* infection as the second most common overall cause of HAIs reported to the National Healthcare Safety Network (NHSN) has also been of a particular concern [12]. Despite the variation in the incidence rates of these infections and the diversity of the causative organisms, broad effective control methods are needed along with comprehensive tracking and monitoring systems that could assess the effectiveness of these interventions [5]. While there has been a significant progress in reducing the standardized infection ratio (SIR) of *S. aureus*, particularly of its resistant strain (MRSA) [5], nosocomial infections continue to plague both medical and residential settings [7]. Additionally, despite employing current methods to control staphylococcal bacteremia [13], serious adverse events among patients remain unsatisfactorily high. A recent review of epidemiological studies on the incidence of MRSA infections has indicated that there is no definitive evidence that the incidence of these infections is declining [5]. The emergence of antibiotic-resistant bacterial strains illustrates pathogens’ persistence despite the use of powerful, highly specific chemical agents and disinfectants.

Viruses are also considered an important source of HAIs. Patients, doctors, nurses, other staff, students, trainees, and visitors could be potential carriers [14]. Specifically, healthcare-acquired viral respiratory diseases are common among children [15]. Nosocomial virus respiratory infections are an underestimated cause of morbidity and mortality, although studies have showed that viruses are responsible for 20% of nosocomial pneumonia [16]. A retrospective study at a tertiary care hospital estimated an incidence of 5 cases/10,000 admissions and 44 cases/10,000 admissions in adult and pediatric patients, respectively [17]. Moreover, the recent emergence of the COVID-19 pandemic, which is caused by the novel coronavirus, SARS-CoV-2, poses a significant risk to healthcare workers in addition to traditional nosocomial infections. The rapid spread of SARS-CoV-2 led to a global outbreak that has been declared as a pandemic by the World Health Organization (WHO). SARS-CoV-2 virus is a member of the Coronaviridae family of viruses, which are enveloped positive-sense single-stranded RNA viruses [18]. SARS-CoV-2 was found to spread via aerosols, large droplets, or contact with infectious secretions. Although the transmission of SARS-CoV-2 in a healthcare setting has not been linked directly to contaminated surfaces, previous experience with other coronaviruses demonstrates that contaminated surfaces pose an important risk of infection transmission within healthcare environments and in the community [19]. The virus has been shown to survive for up to three hours in aerosols, up to four hours on copper, up to 24 h on cardboard, and up to two to three days on plastic and stainless steel surfaces [19]. Recently, a study showed that with initial viral loads that are equivalent to the highest titers excreted by infectious patients, the virus was isolated viable for up to 28 days at 20 °C from common surfaces such as glass and stainless steel [20]. Environmental contamination with aerosolized droplets containing this pathogen can serve as a reservoir for infection and must be controlled by effective disinfection protocols. Minimizing the degree of environmental contamination with highly effective decontamination measures would aid in the overall containment efforts of the COVID-19 pandemic.

Several studies demonstrated that environmental cleaning and surface disinfection strategies reduce contamination and transmission of infections [21]. However, traditional disinfection methods such as chlorination could produce harmful byproducts that could cause toxic adverse events [22]. The most common side effects of using traditional disinfectants according to the Centers for Disease Control and Prevention (CDC) are eye irritation (55%), followed by neurologic manifestations such as headache and dizziness (32%), respiratory symptoms such as throat irritation and cough (30%), and skin irritation (24%) [23]. Water pathogens also pose a health problem and are considered a source for HAIs that are caused by *Legionella* and *Mycobacterium* species [24]. Disinfection byproducts resulting from water chlorination are found to be associated with health hazards and increased risk for bladder cancer [25]. There is also a trend toward positive association with small gestational age, intrauterine growth restriction, preterm delivery, and higher frequency of congenital anomalies [25,26].

Due to the efforts of Medicare to encourage improvements in patient safety in hospitals and other healthcare facilities, these facilities have improved their screening and anti-microbial efforts, including microbial surveillance, testing for antimicrobial resistance [27], and introduction of comprehensive programs aimed at reducing contamination and cross-transmission. Such efforts are further encouraged to employ multiple simultaneous cleaning measures [28]. The study done by the latter group used multiple detergents, disinfectants, and new technologies, such as ultraviolet disinfection. The results of the outcome questionnaires indicate an incremental benefit over other traditional approaches. Therefore, further studies that aim at identification and validation of alternative effective, safe, and environmentally friendly methods are needed.

Several new disinfection technologies have recently emerged for infection control in hospitals and the community [22]. These technologies include new disinfectant liquids such as improved hydrogen peroxide liquids, electrolyzed water, and cold air atmospheric pressure plasma systems [22]. Additionally, self-disinfecting surfaces that are coated with metals, such as copper and silver with innate antimicrobial properties, have been used to prevent the growth of bacteria on surfaces in hospitals [22]. Most recently, photocatalytic disinfection strategies have been developed and showed potential applications that could solve significant health and environmental problems [29]. Photocatalytic technology commonly involves a titanium dioxide semiconductor, which is generally safe, chemically stable, economic, and allows repeated use without substantial loss of its catalytic ability [29]. The antimicrobial effects of photocatalysis are mainly mediated by the induction of air ionization, which produces free electrons and positive ions leading to the formation of the superoxide radical anion O^−2^, which forms hydrates. These intermediate species, which are called “cluster ions,” react rapidly with particulates and volatile organic species. The mechanism of their bactericidal activity is mediated by a combination of cell membrane damage and induction of oxidation of internal cellular components [29].

In this study we aimed to test the efficacy of photocatalytic reactors in reducing hospital rooms contamination with *S. aureus* and *C. difficile* strains. Moreover, we validated the use of the photocatalytic reactor to inactivate enveloped RNA viruses using dengue virus type 2 (DENV2) as an experimental model [30]. The antiviral efficacy of the photocatalytic reactor was determined based on reduced or complete loss of viral infectivity in a cell culture system following exposure of the viral samples to the photocatalytic reactor treatment.

## 2. Experimental Section

### 2.1. Assessment of the Effect of the Photocatalytic Reactor on S. aureus and C. difficile

**Figure 1:**
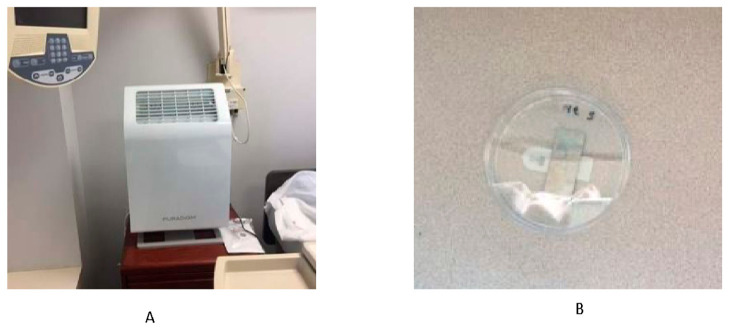
The photocatalytic reactor setup in the hospital room. (**A**) The photocatalytic reactor. (**B**) Sterile stainless steel carriers were individually placed in sterile petri dishes and affixed to the bottom of each petri dish with adhesive and placed at multiple locations.

Institutional review board (IRB) approval was not required for this study, which was performed in unoccupied patient rooms. Experiments that involved *S. aureus* were conducted at Pontiac General Hospital (Pontiac, MI, USA) and National Sanitation Foundation (NSF) International laboratories (Ann Arbor, MI, USA). *S. aureus* (ATCC, BAA-41, Manassas, VA, USA) was cultured from a freezer stock. A total of 100 µL freezer stock was transferred into 10 mL trypticase soy broth (TSB) and incubated for 18–24 h at 35 ± 1 °C. The bacterial culture was vortexed and allowed to settle for 15 min, then the upper two thirds of settled culture were removed. The culture was then centrifuged twice at 3500× *g* for 15 min and re-suspended in 10 mL of 0.1% peptone water. After that, the cell suspension was diluted in 0.1% peptone water to an organism density of approximately 7.5 × 10^5^ colony forming unit (CFU)/mL. The cell density (CFU/mL) was calculated by plating serial dilutions onto Petrifilm Aerobic Count Plates followed by incubation for 18–24 h at 35 ± 1 °C. A stainless steel tray was rinsed with 95% ethanol and then rinsed with deionized water and air-dried. Stainless steel carriers were dipped in 95% ethanol using forceps, rinsed with deionized water, and air-dried. Carriers were then placed in the stainless steel tray, covered with aluminum foil, and autoclaved on dry waste cycle at 121 °C for 30 min. The sterile carriers were individually placed in sterile petri dishes and affixed to the bottom of each petri dish with a sterilized adhesive (Command Brand Damage Free Hanging Strips, Figure 1B). Fourteen carriers were inoculated with 10 µL of the 7.5 × 10^5^ CFU/mL cell suspension. A single droplet was applied without spreading. One additional sterility control carrier with only 10 µL sterile 0.1% peptone water was also prepared. All carriers were allowed to dry for 20–40 min under conditions of 20–25 °C and 40–45% humidity.

Experiments that involved *C. difficile* were conducted at Insight Institute of Neurosurgery and Neuroscience (Flint, MI, USA) and the NSF International laboratories (Ann Arbor, MI, USA). *C. difficile* (ATCC, 43598, Manassas, VA, USA) spores were obtained from the NSF International laboratories; they were prepared based on the published Environmental Protection Agency (EPA) MB-28 protocol [31]. Spore suspension was serially diluted in phosphate-buffered saline (PBS) and plated in duplicate to the Brain Heart Infusion Agar with Horse Blood and Taurocholate agar plates (BHIY–HT) and incubated anaerobically for 120 ± 4 h at 36 ± 1 °C after which the CFU/mL values were calculated. Stainless steel carriers were sterilized prior to use by dipping them in 70 to 95% ethanol, rinsing them with deionized water, and then autoclaving them at 121 °C. Carriers were then aseptically transferred into sterile petri dishes. Eight carriers were inoculated by 10 µL of inoculum suspension (3.3 × 10^5^ CFU/mL) each and were then air-dried in a biosafety cabinet for one hour. The samples were then aseptically transferred into sterile conical tubes and transported under cold conditions to the testing rooms. Two additional sterility control carriers with only 10 µL sterile 0.1% peptone water were prepared.

### 2.2. Onsite Hospital Room Testing

The stainless steel carriers containing *S. aureus* (ATCC, BAA-41) in the planktonic form were placed in two unoccupied rooms with similar air volume and airflow. One of these rooms served as a control, with no photocatalytic treatment. Eleven carriers were placed in the testing room and three were placed in the control room. Each room contained one attached bathroom with the door that remained closed during testing. Each room contained one large window which remained closed during testing with the blinds left open and lights remained off during testing. Doors remained closed throughout the period of testing. One photocatalytic reactor (Puradigm, Las Vegas, NV, USA) was placed in the testing room for 24 h prior to the samples and then maintained for 24 h during the testing (Figure 1A and Supplementary 1). The stainless steel coupons inoculated with *S. aureus* were placed at various locations all over the test and control rooms, with focus on high-touch areas, including the patient bed, vanity, bedside table, windowsill, footboard, room door handle, television remote control, and light switches (Supplementary 1). All coupons were collected after 24 h and returned to the laboratory for processing. Each one was placed into a 50 mL centrifuge tube containing 30 mL of TSB and vortexed for 15 s. Then, 10^−1^ dilutions of each sample were plated in duplicate onto Petrifilm Aerobic Count Plates and incubated for 18–24 h at 35 ± 1 °C, after which CFU/mL values for each sample were calculated.

The stainless steel carriers containing *C. difficile* spores were placed in two unoccupied rooms with similar air volume and airflow. One of these rooms served as a control and one as a treatment room. A test chamber was constructed in each of the testing and control rooms by wrapping a plastic sheeting around a table and sealing the seams with duct tape (Supplementary 1). The photocatalytic reactor (Puradigm, Las Vegas, NV, USA) was placed in the testing chamber for 24 h prior to the samples and then maintained for 48 h for the duration of the treatment. Five stainless steel carriers were placed vertically in the secured chamber and exposed to the treatment for 48 h, while three coupons were placed in the control room with no exposure to treatment. Longer duration of treatment (48 h) was considered due to the known resistance of *C. difficile* spores compared to MRSA [32]. Each carrier was then aseptically placed into 30 mL PBS containing 0.1% Tween 80 (PBS-T) in a 50 mL conical tube. Each tube was vortexed for 30 s and the eluent was serially diluted in PBS, spread in duplicate onto BHIY-HT, and incubated anaerobically for 120 ± 4 h at 36 ± 1 °C, after which CFU/mL values for each sample were calculated.

### 2.3. Propagation of Viruses and Cell Culture

Experiments that included viruses were conducted at Central Michigan University. DENV2 strain New Guinea was obtained from the Connecticut Agricultural Experiment Station. The virus was passaged in C6/36 cells and the stocks were generated 8 days post-infection by harvesting cell-free supernatants. The cells were maintained in DMEM containing 10% fetal bovine serum, tryptose phosphate, and antibiotics at 30 °C. DENV2 was grown to approximately 2 × 10^3^ focus forming units (FFU) per mL. FFU assays were performed using Aag2 cells. Each of these reagents were provided by Erol Fikrig at Yale University School of Medicine. Both C6/36 and Aag2 cells were maintained in high-glucose DMEM (Gibco, Waltham, MA, USA) with 10% fetal bovine serum (FBS), 5% penicillin–streptomycin (ThermoFisher, Waltham, MA, USA), and 5% tryptose phosphate (ThermoFisher, Waltham, MA, USA) at 28 °C with 5% CO_2_.

### 2.4. Viral Inactivation Assay

#### 2.4.1. Inside a Biosafety Cabinet Experiment

Approximately 30 FFU of DENV2 were added to triplicate wells of two tissue culture-treated plastic 96-well plates. The first plate was placed inside of a biosafety cabinet along with the photocatalytic reactor (Puradigm, Las Vegas, NV, USA). The photocatalytic reactor was activated while the biosafety cabinet sash was closed. The second plate was placed in the second room at least 20 feet away from the closed biosafety cabinet. Six hours post-treatment, the triplicate DENV2 samples were transferred to monolayers of Aag2 cells and incubated for one hour. Unbound virus was removed and the cells were maintained in fresh media for 3 days. The cells were then fixed with 4% paraformaldehyde (Sigma, St. Louis, MO, USA), permeabilized with 0.1% Triton X (Sigma, St. Louis, MO, USA), and then stained with anti-dengue virus antibody 3H5.1 (MilliporeSigma, Burlington, MA, USA), followed by treatment with an anti-mouse IgG-HRP secondary antibody (Abcam, Cambridge, UK) and addition of a peroxidase substrate (Vector Laboratories, Burlingame, CA, USA). The number of foci was counted manually using an Evos XL Core Cell Imaging System.

#### 2.4.2. Outside a Biosafety Cabinet Experiment

Approximately 150 FFU of DENV2 were added to triplicate wells of five tissue culture-treated plastic 96-well plates. Four plates were opened and placed inside of a biosafety cabinet. The biosafety cabinet was powered off while the sash was left open. The photocatalytic reactor (Puradigm, Las Vegas, NV, USA) was placed on the ground in the lab, outside the biosafety cabinet, activated, and set to the second power setting. The room dimensions were 14 feet in length, 9 feet in height, and 10 feet in width. The fifth plate was opened and placed in a drawer in the second room at least 20 feet away from the biosafety cabinet. Plates in the biosafety cabinet were removed and placed along with the fifth plate for 6, 12, 18, and 24 h post-treatment. Triplicate DENV2 samples were resuspended in 100 µL cell culture media by pipetting up and down ten times, and then the virus was transferred to monolayers of Aag2 cells and incubated for one hour. Unbound virus was removed and the cells were maintained in fresh media for 3 days. The cells were then fixed with 4% paraformaldehyde (Sigma, St. Louis, MO, USA), permeabilized with 0.1% Triton X (Sigma, St. Louis, MO, USA), and then stained with anti-DENV2 antibody 3H5.1 (Millipore Sigma, Burlington, MA, USA), followed by treatment with an anti-mouse IgG-HRP secondary antibody (Abcam, Cambridge, UK) and addition of a peroxidase substrate (Vector Laboratories, Burlingame, CA, USA). The number of foci was counted manually using an Evos XL Core Cell Imaging System.

### 2.5. Data Analysis

Statistical analyses were performed using SPSS v25 (IBM, Armonk, NY, USA). Log number of *S. aureus* and *C. difficile* per exposed and unexposed samples (log CFU/carrier) were calculated. Percentages of reduction and log reductions of *S. aureus* and *C. difficile* (CFU/mL) after 24 h- or 48 h-exposure, respectively, were calculated. Descriptive statistics and two-sample *t*-test were used to compare the log CFU/carrier values of the treated and untreated samples after the duration of treatment in each experiment. For the virus experiment, descriptive statistics and multiple two-sample *t*-tests were used to compare the average FFU/mL values between the treated and control samples in each group. Percentages of reduction in infectivity (FFU/mL) after 6, 12, 18, and 24 h of treatment compared to the control were calculated. The alpha level was set at *p* < 0.05 for all analyses.

## 3. Results

### 3.1. The Photocatalytic Reactor Reduces Growth of S. aureus and C. difficile Cultures in Hospital Rooms

**Figure 2:**
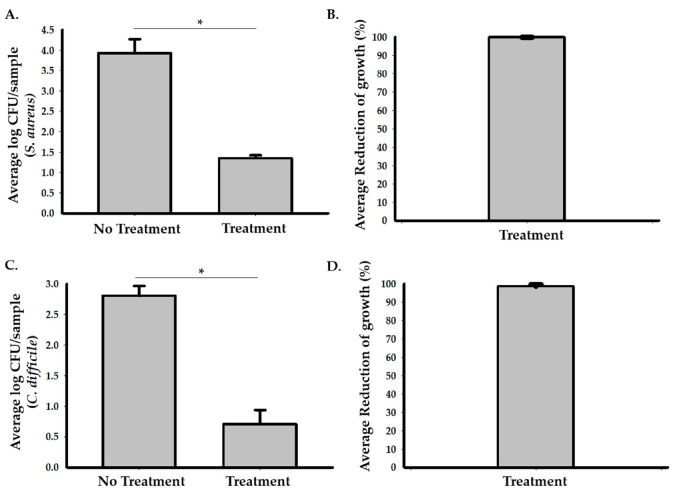
The photocatalytic reactor treatment reduces growth of *S. aureus* and *C. difficile* in hospital rooms. (**A**) Eleven samples containing *S. aureus* were placed in the testing room and three samples were placed in the control room. The graph represents the average log CFU/carrier values in the testing room compared to the control. The Student’s *t*-test analysis data showed that there was a significant reduction in the log CFU of *S. aureus* in the testing room after 24 h of treatment (M = 1.35, SD = 0.22) compared to the control (M = 3.92, SD = 0.60, * *p* < 0.001). (**B**) A representative graph for the percentage of *S. aureus* growth reduction in the testing room compared to the control. There was an average 99.86% (95% CI: 99.8 to 99.9%) reduction in bacterial growth in the testing room compared to the control. (**C**) Five samples containing *C. difficile* spores were placed in the testing room and three samples were placed in the control room. The graph represents the average log CFU/carrier values of the samples in the testing room compared to the control. Two sample *t*-test analysis data showed that there was a significant reduction in the log CFU of *C. difficile* in the testing room after 48 h of treatment (M = 0.7, SD = 0.51) compared to the control (M = 2.8, SD = 0.28, * *p* < 0.001). (**D**) A representative graph for the percentage of *C. difficile* reduction in the testing room compared to the control. There was an average 99.2% (95% CI: 98.1 to 99.7%) reduction in bacterial growth in the samples that were placed in the testing room compared to the control.

To assess the efficacy of photocatalysis in reducing the growth of *S. aureus* in a hospital room, we used stainless steel carriers inoculated with 10 µL of the 7.5 × 10^5^ CFU/mL of *S. aureus* and placed the samples at multiple locations in an unoccupied patient room (Figure 1 and Supplementary 1). A hospital room with no exposure to the photocatalytic reactor was used as a control. Based on the calculated CFU of each sample (Figure 2A,B and Supplementary 1), the exposure to the photocatalytic reactor for 24 h significantly inhibited the growth of *S. aureus* in the hospital room and resulted in the 99.86% (95% CI: 99.8 to 99.9%) average reduction in bacterial growth compared to the control room. Two-sample *t*-test analysis showed that the log CFU of *S. aureus* in the testing room (M = 1.35, SD = 0.22) was significantly smaller (*p* < 0.001) compared to the controls (M = 3.92, SD = 0.60), with the 2.9 (95% CI: 2.77 to 3.03) average log reduction in *S. aureus* samples in the testing room.

The efficacy of photocatalysis in reducing the growth of *C. difficile* was also assessed in unoccupied rooms. We used stainless steel carriers containing *C. difficile* spores and placed them inside test chambers in treatment and control rooms. Our results (Figure 2C,D and Supplementary 1) showed that the growth of *C. difficile* was significantly reduced in the treated samples compared to the control samples. The average percentage of reduction in growth was 99.2% (95% CI: 98.1 to 99.7%) after 48 h of treatment compared to the control. Two sample *t*-test analysis showed that the log CFU of *C. difficile* in the testing room (M = 0.71, SD = 0.51) was significantly smaller (*p* = 0.0003) compared to the controls (M = 2.8, SD = 0.28), with the 2.1 (95% CI: 1.71 to 2.58) average log reduction in growth in the testing room.

### 3.2. The Photocatalytic Reactor Reduces the Infectivity of Dengue Virus Type 2

**Figure 3:**
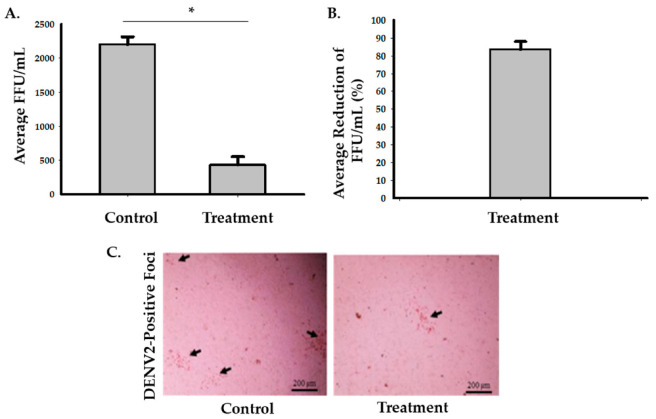
Inactivation of DENV2 with the photocatalytic reactor following 6-h exposure in a biosafety cabinet. (**A**) Viral growth assessed after the viral samples were treated with photocatalysis and inoculated into Aag2 cells. Three samples were used in each group. The Student’s *t*-test analysis showed that there was a significant reduction (* *p* < 0.05) in the average FFU/mL value in the treated samples (M = 433, SD = 208) compared to the control (M = 2200, SD = 200, *p* = 0.0004). (**B**) A representative graph for the average percentage of reduction in FFU/mL values after six hours of exposure to the photocatalytic treatment which was 83.5% (95% CI: 74.5 to 92.5%). (**C**) Representative digital images of DENV2-positive foci from the control and treated samples were taken at 20× magnification using an Evos Core inverted microscope.

**Figure 4:**
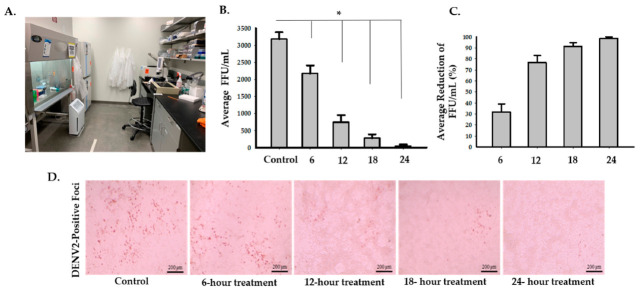
Inactivation of DENV2 with the photocatalytic reactor outside the biosafety cabinet at 6, 12, 18, and 24-h timepoints. (**A**) Image of the photocatalytic reactor placed on the ground next to a biosafety cabinet. Four 96-well plates (three samples in each one) in total were placed inside the biosafety cabinet in the testing room. The fifth 96-well plate containing three samples was placed in the control room. Plates in the biosafety cabinet were removed and placed along with the fifth plate 6, 12, 18, and 24 h post-treatment. (**B**) DENV2 viral growth after exposure to the photocatalytic reactor treatment for 6, 12, 18, and 24 h compared to the control. Multiple Student’s *t*-test analyses of each treatment and control groups showed that there were significant reductions (* *p* < 0.05) in infectivity of DENV2 in the treated samples (*p* = 0.0326 (M = 2173, SD = 412), *p* = 0.001 (M = 747, SD = 356), *p* = 0.0002 (M = 280, SD = 191), and *p* = 0.0001 (M = 47, SD = 81) after 6, 12, 18, and 24 h, respectively) compared to the control (M = 3180, SD = 354). (**C**) A representative graph for average percentages of reduction in infectivity which were 31.7% (95% CI: 17 to 46.4%), 76.5% (95% CI: 63.8 to 89.2%), 91.2% (95% CI: 84.4 to 98%), and 98.5% (95% CI: 95.7 to 100%) after 6, 12, 18, and 24 h of treatment, respectively. (**D**) Representative digital images of DENV2-positive foci from the control and treated samples were taken at 20× magnification using an Evos Core inverted microscope.

To assess the effects of the photocatalytic reactor on enveloped RNA viruses, we used DENV2 as an experimental model. There was an average of 83.5% (95% CI: 74.5 to 92.5%) decrease in infectivity after 6 h of treatment inside of the biosafety cabinet (Figure 3 and Supplementary 2). Two-sample *t*-test analysis showed that exposure of DENV2 to the photocatalytic reactor inside a biosafety cabinet for 6 h significantly reduced the number of FFU/mL compared to the control (*p* = 0.0004). The second set-up experiment, while having the photocatalytic reactor inside the room but outside the biosafety cabinet, showed that the average percentage of reduction was 31.7% (95% CI: 17 to 46.4%), 76.5% (95% CI: 63.8 to 89.2%), 91.2% (95%: CI 84.4 to 98%) and 98.5% (95% CI: 95.7 to 100%) after 6, 12, 18, and 24 h, respectively (Figure 4 and Supplementary 2). Multiple two-sample *t*-test analyses revealed that there were significant decreases in DENV2 infectivity in the treated samples (*p* = 0.0326, *p* = 0.001, *p* = 0.0002, and *p* = 0.0001 after 6, 12, 18, and 24 h, respectively) compared to the control (Figure 4 and Supplementary 2).

## 4. Discussion

Contaminated surfaces and environments are an important source of HAIs and infection spread within hospital and non-hospital facilities [22]. Multidrug-resistant bacteria have both economical and health impacts, and it is estimated that by 2050, they will be held responsible for 10 million deaths [33,34]. Surfaces that are not properly cleaned or disinfected facilitate the transmission of communicable diseases among patients, their families, and the healthcare workers [1]. Despite the use of more effective cleaning and disinfection strategies, the abundance of infective agents is on the rise in many hospital areas including patients’ rooms and frequently touched surfaces [27].

Surface decontamination is a basic approach to reduce the spread of infections. With the significant burden of *C. difficile* and MRSA infections within hospitals and the emergence of highly transmissible pandemic diseases such as COVID-19, there is a pressing need for efficient surface cleaning and decontamination technologies [1,35]. Our results demonstrated a significant reduction in *C. difficile* and *S. aureus* growth in the hospital rooms that were treated with a photocatalytic reactor compared with the control room. Moreover, we tested the efficacy of the photocatalytic reactor in suppressing the infectivity of RNA viruses using DENV2 as the experimental model. Our results demonstrated that the exposure of viruses to the photocatalytic reactor treatment led to a statistically significant and dose-dependent decrease in viral infectivity.

The photocatalytic reactors involve a photocatalytic oxidation technology that has been shown to reduce the growth of the microbial organisms on contaminated surfaces and in the surrounding environment [29]. Previous research showed that the antimicrobial mechanism of photocatalytic oxidation depends on the generation of “cluster ions” within the environment. The primary positive ions dinitrogen (N_2_), dioxygen (O_2_), nitrogen (N), and oxygen (O) are very rapidly converted, within microseconds, to protonated hydrates, H^+^ (H_2_O), while free electrons quickly attach to oxygen to form the superoxide radical anion O^−2^, which also can form hydrates [36]. Ionization of oxygen and formation of reactive oxygen species (ROS) is involved in many applications of air ionization. Previous study showed that superoxide ion clusters that are formed in the air could react rapidly with airborne particulates. ROS react with volatile organic compounds (VOCs) and yield particulates that are more easily removed or tolerated [37].

Several new ultraviolet-based technologies have been reported to effectively control and eliminate airborne contaminants. These include controlling aerosol contaminants and airborne fungal spores and bacteria [38]. Ultraviolet light is used as an internal light source in photocatalytic reactors. Photocatalysis is a branch of chemistry which deals with chemical reactions taking place in the presence of light and a photocatalyst. A photocatalyst is a semiconductor with the electrical conductivity value that falls between that of a conductor and an insulator [39]. In many photocatalytic systems, the semiconductor is composed of titanium dioxide that might be combined with other elements [40]. This combination can produce an ionized cloud of bactericidal and antiviral molecules. When ultraviolet energy strikes the titanium dioxide, it triggers a catalytic reaction that may produce the desired cloud of bactericidal molecules in the air [40]. These molecules, upon contact with any bacteria, mold, or viruses, may cause a robust antimicrobial effect. Semiconductor photocatalysis, with a focus on titanium dioxide as a durable photocatalyst, has been used for potable water treatment, air purification, destruction of microorganisms, inactivation of cancer cells, odor control, photo splitting of water to produce hydrogen fuel, fixation of nitrogen, and clean-up of oil spills [38].

Plethora of evidence showed that photocatalysis can kill and inactivate a lot of microorganisms from different kingdoms. The efficacy of photocatalytic disinfection using titanium dioxide was previously studied on gram-negative bacteria including mainly *E. coli*, *Acinetobacter*, *Legionella pneumophila*, and *Pseudomonas aeruginosa* [41,42,43,44,45,46]. Although gram-positive bacteria were shown to be more resistant than the gram-negative ones [41,47,48], studies on MRSA, *S. aureus*, and *Listeria monocytogenes* showed that they are vulnerable to killing by photocatalysis [49,50,51]. Dunlop and colleagues also showed that photocatalytic coated surfaces reduce viability of *C. difficile* spores and cells within biofilms [32]. Interestingly, studies on viruses including SARS, influenza A/H1N1, norovirus, influenza A/H_3_N_2_ also showed that photocatalysis has effects on them [52,53,54,55]. Additionally, it was found that photocatalysis inactivates microbial toxins [56,57,58].

The mechanism by which photocatalysis kills microbial cells was mainly studied in *E. coli* [59]. An early suggested mechanism was that oxidation process decreases coenzyme A levels in cells which lead to metabolic reduction [60,61]. Recent studies have shown that the ROS generated by photocatalysis induce lipid peroxidation of bacterial cell membranes causing increased membrane permeability and cell membrane damage [59]. A strong positive association between potassium leakage, which is caused by increased cell permeability, and bacterial inactivation suggested that depletion of potassium has a main effect on bacterial cells [49]. Moreover, the permeability of cells has been found to be too extensive, allowing even large macromolecules such as beta-D–galactosidase molecules [62]. Additionally, direct effect on intracellular compartments were also noted with observed reduction in enzymatic activity [62]. ROS also trigger oxidative stress within the microorganism causing diminished ability to produce adenosine triphosphate (ATP) [59].

The effect of photocatalysis on the cell wall and membrane was also studied using high resolution image techniques. Kangwansupamonkon and colleagues performed scanning electron microscope (SEM) studies on *S. aureus*, MRSA, *E. coli*, and *Micrococcus luteus* and observed morphological alterations in the cell wall and outer membrane [63]. Cell membrane bubbles and vesicle-containing material detached from the cells were seen also in *P. aeruginosa* examined by SEM and transmission electron microscopy (TEM) [64,65]. Interestingly, the morphological changes that were seen with photocatalytic treatment were similar to those of antibiotic aminoglycosides [65]. Imaging techniques also help in understanding the sequence of events. Utilizing atomic force microscopy, Sunada and colleagues showed that the outer cell membrane was disrupted first, followed by the cytoplasmic membrane, ultimately leading to cell death [66]. Similar membrane damage was also observed in fungi and members of Kingdom Protozoa, such as *Oedogonium* species, *Chroococcus* species, *Candida albicans*, *Giardia lamblia*, and *Tetrahymena pyriformis* [29]. A study on viruses also showed that the photocatalytic treatment of influenza virus A/H1N1 resulted in significant structural damage that involved damage to the H and N projections [53].

## 5. Conclusions

Our results showed that the photocatalytic reactor treatment reduced growth of *S. aureus* and *C. difficile,* and decreased the infectivity of DENV2. This study was limited to only one bacterial strain of each of *C. difficile* and *S. aureus* in addition to an RNA virus (DENV2) as a proof of concept. Our data demonstrate that there is a potential efficacy of using photocatalytic reactors to clean surfaces and environments within hospitals, laboratories, and non-hospital facilities which may reduce spread of infections. Future studies should aim at testing multiple pathogens with focusing on mechanisms and safety and modifying variable factors to optimize the efficacy of photocatalytic treatment. This will further validate the photocatalytic reactor treatment as a method to reduce the spread of bacterial and viral infections and improve containment of pandemics such as COVID-19 among hospital workers, patients, and the community.

## Data Availability

Data is contained within the article or supplementary material.

## Supplementary Materials

The following are available online at https://www.mdpi.com/2036-7449/13/1/8/s1.
